# Node retraction during patterning of the urinary collecting duct system

**DOI:** 10.1111/joa.12239

**Published:** 2014-10-07

**Authors:** Nils O Lindström, C-Hong Chang, M Todd Valerius, Peter Hohenstein, Jamie A Davies

**Affiliations:** 1 Roslin Institute, University of EdinburghEaster Bush, UK; 2 Centre for Integrative Physiology, University of EdinburghEdinburgh, UK; 3 Department of Surgery, Transplant Institute, Beth Israel Deaconess Medical CenterBoston, MA, USA

**Keywords:** branching morphogenesis, collecting duct, development, modelling, embryonic kidney, organogenesis, pattern formation, ureteric bud

## Abstract

This report presents a novel mechanism for remodelling a branched epithelial tree. The mouse renal collecting duct develops by growth and repeated branching of an initially unbranched ureteric bud: this mechanism initially produces an almost fractal form with young branches connected to the centre of the kidney via a sequence of nodes (branch points) distributed widely throughout the developing organ. The collecting ducts of a mature kidney have a different form: from the nephrons in the renal cortex, long, straight lengths of collecting duct run almost parallel to one another through the renal medulla, and open together to the renal pelvis. Here we present time-lapse studies of E11.5 kidneys growing in culture: after about 5 days, the collecting duct trees show evidence of ‘node retraction’, in which the node of a ‘Y’-shaped branch moves downwards, shortening the stalk of the ‘Y’, lengthening its arms and narrowing their divergence angle so that the ‘Y’ becomes a ‘**V**’. Computer simulation suggests that node retraction can transform a spread tree, like that of an early kidney, into one with long, almost-parallel medullary rays similar to those seen in a mature real kidney.

## Introduction

This report describes a novel morphogenetic mechanism within the branching epithelial tubule system of the kidney. The mechanism – retrograde retraction of nodes (branch points) – may be important in transforming an immature, fractal-like collecting duct tree into the characteristic almost parallel, radial arrangement of medullary collecting ducts seen in the mature organ.

The mature mouse kidney is organized in concentric zones. The outer zone, the cortex, contains the glomeruli and nephrons that filter blood and recover valuable solutes. Nephrons drain into a tree-like system of urinary collecting ducts. These fine tubes run radially down through first the outer medulla (which also contains the loops of Henle descending from the nephrons) and then the inner medulla, to drain into the renal pelvis, which in turn empties into the ureter. The histology of the medulla is dominated by the almost-parallel tubes of the collecting system, which converge at the papillae at the edge of the renal pelvis.

The collecting duct system develops by repeated branching of an initially unbranched epithelial tube, the ureteric bud (Remak, 1855). The branching process is induced by paracrine signals from the surrounding mesenchymal cells, particularly glial cell line-derived neurotrophic factor (Sainio et al. 1997), neurturin (Davies et al. 1999), and fibroblast growth factors (Trueb et al. 2013). It also requires specific molecules of the extracellular matrix such as nidogen, fibronectin and Ecm1 (Ekblom et al. 1994; Ye et al. 2004; Paroly et al. 2013), and receptors and intracellular signal transduction pathways associated with matrix signalling (Zhang et al. 2009; Lee et al. 2010; Tai et al. 2013).

The cell biology that drives branching morphogenesis remains incompletely understood. The ureteric bud tree is divided into ‘tip’ and ‘stalk’ regions, distinguishable by gene expression (Lin et al. 2001; Kispert et al. 1996; Michael et al. 2007). Most cell proliferation takes place in the tip (Michael & Davies, 2004), which acts as a stem cell population, maintaining and expanding itself and giving rise to stalk cells (Shakya et al. 2005). Formation of branch points requires both mitosis (Michael & Davies, 2004) and cytoskeletal activity (Michael et al. 2005; Meyer et al. 2006; Kuure et al. 2010). Branching is dominated by bifurcation, but trifurcations and lateral branches can also be seen in time-lapse studies (Watanabe & Costantini, 2004). Careful analysis of branch points reveals that newly diverging tips proceed at relative angles of about 90° (Watanabe & Costantini, 2004).

The rich understanding obtained from these studies of early ureteric bud/collecting duct branching morphogenesis must, however, be incomplete because repeated branching at 90° could not by itself produce the anatomy of the mature system. In particular, it fails to account for two features: it would not produce the long, straight collecting ducts that run almost parallel through the medulla, and it would not have large numbers of tubules converging at the papillae rather than meeting in a spaced, sequential way all the way down a tree (Kim et al. 2002). Suggested explanations for the existence of long medullary ducts have included selective longitudinal growth, in particular by orientated cell division, and convergent extension movements (Cebrián et al. 2004; Yu et al. 2009; Costantini & Kopan, 2010; Costantini, 2012). Convergence at the medulla is more difficult to explain this way: this has generally been assumed to result from an expansion of the renal pelvis in a way that obliterates early branch points (Potter, 1972).

Here, we report evidence for an additional mechanism of collecting duct remodelling that is revealed by time-lapse observation of kidney rudiments developing in culture for 3–12 days. This mechanism, node retraction, moves the node (branch point) of a ‘Y’-shaped branch downwards, so that the stalk of the ‘Y’ shortens and the arms lengthen and narrow their divergence angle to make a ‘**V**’. This retraction takes place in absolute terms, not just relative to the expanding diameter of the kidney. When it happens in neighbouring branches, its effect is to make them appear to converge on one place, as real branches do at a papilla. We propose that node retraction, in conjunction with the growth mechanisms already discovered, is an important mechanism in the remodelling of a spread ureteric bud tree into the radially organized mature collecting duct system.

## Materials and methods

### Kidney culture for still images

For Fig. 6A, kidney rudiments were isolated by mechanical dissection from E11.5 wild-type mouse embryos, in Earle's modification of Eagle's minimum essential medium (Sigma M5650). They were cultured on track-etched polycarbonate filters supported by a Trowell grid at the surface of culture medium (M5650 with 10% fetal calf serum), at 37 °C in 5% CO_2_. The rudiments were fixed by replacement of the medium with methanol, initially at −20 °C and allowed to warm to room temperature over 15 min. The rudiments were washed in PBS for 15 min, stained overnight in 1/100 (AbCam), washed in PBS 8 h, stained overnight in FITC anti-Mouse IgG (Sigma), washed in PBS and anti-calbindin-D28k mounted in 50 : 50 glycerol : phosphate-buffered sale (PBS) between 64 × 22 mm coverslips that are themselves separated by 22 × 22 mm coverslips to prevent the samples being crushed.

### Kidney culture for time-lapse images

For time-lapse imaging for Figs 3 and Supporting Information Movies S1, S2 and S3, E11.5 kidneys were isolated from Lgr5-EGFP-ires-CreERT2 knock-in embryos (Barker et al. 2007). Each isolated kidney was cultured in the Sebinger system described in Sebinger et al. (2010) and Chang & Davies (2012), in humidified 5% CO_2_/air at 37 °C. After 7 days, the culture was placed on an in-incubator microscope (LumaScope model 500, Etaluma). Images were captured every 15 min for 57 h.

**Fig. 1:**
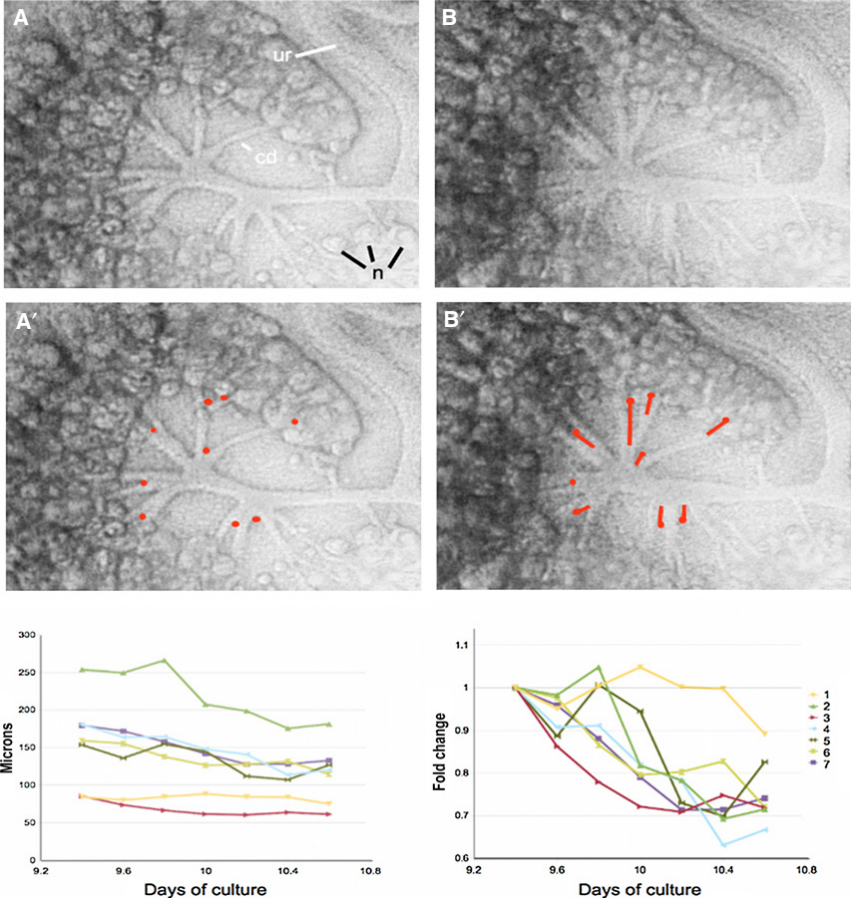
Node retraction in Sebinger culture, seen at low magnification to provide context for the higher magnification images in Figs 2 and 3. (A) and (A’) show an E11.5 kidney cultured for 9.4 days in low-volume culture: the underlying biological image is the same, but spots have been placed on image (A’) to indicate the starting positions of retracting nodes. (B) and (B’) show the same culture 1.2 days later. Some nodes have not moved but those indicated by spots in (A’) have retracted in (B), as shown by the arrows in (B’), which extend from the original position to the final one. In (A), ‘ur’ = ureter, ‘cd’ = collecting duct, ‘n’ = immature nephrons. The graphs show the changing length of seven medullary internodes (Identified in KeyS1) over time in both absolute terms and relative (fold change). Scale: images are 1.07 mm wide.

**Fig. 2:**
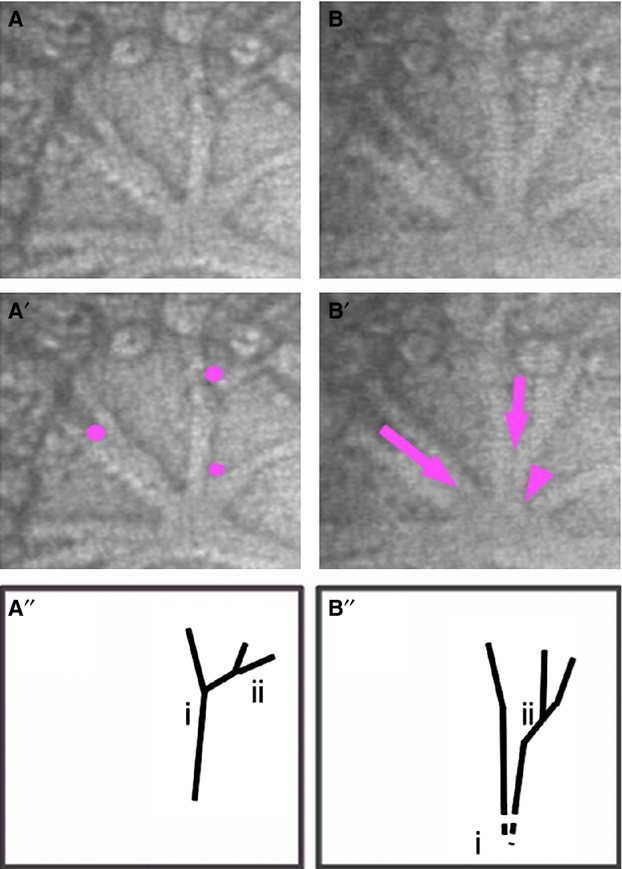
High-magnification view of a portion of the kidney shown in Fig. 1. Again, (A), (A′) and (A″) are of an E11.5 kidney at 9.4 days of culture, and (B), (B′) and (B″) 1.2 days later. Frames (A,B) show anatomy unobstructed by labels, (A′) indicates the position of three nodes undergoing retraction and (B′) indicates, with arrows, the extent of retraction. Images (A″) and (B″) show skeletonized versions of one of the branch systems in (A) and (B) to emphasize how retraction has narrowed the angle of node i. Scale: each image is 161 μm across.

**Fig. 3:**
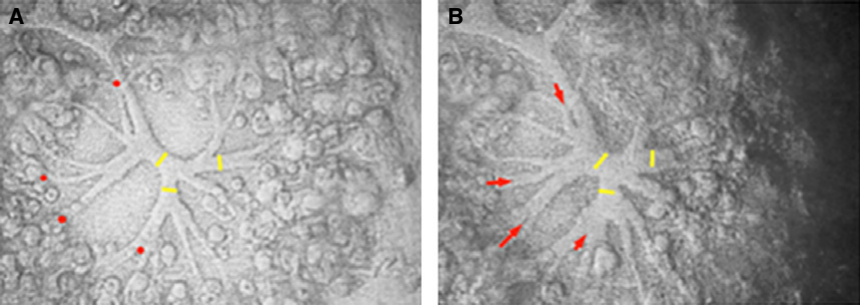
Low-magnification view showing an E11.5 kidney cultured for 9.4 (A) and 1.2 days later (B). The images illustrate both node retraction, shown as before in starting position markers in (A) and movement arrows in (B), and also expansion in girth of the older branches. The yellow bars were drawn to fit exactly across the tubules in (A), and were then copied on to image (B): they now fall about 20% short of spanning the tubules. Scale: each image is 1.27 mm wide.

**Fig. 4:**
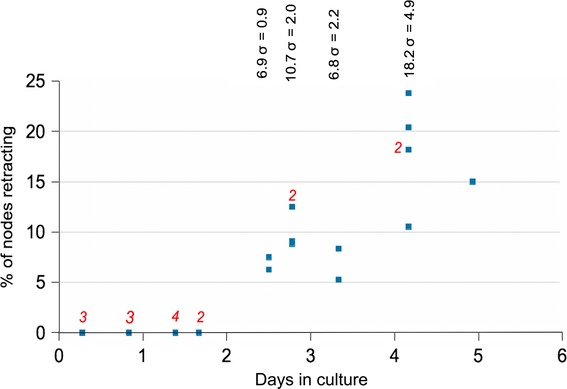
Incidence of node retraction over time in kidneys cultured using the conventional (Saxen) method. Seven kidneys were set up in culture at E12.5–E12.75 and time-lapse images were made of them. At the intervals of culture shown in the graph (chosen, for each kidney, to avoid any periods in which there was any drift of the whole image or any optical problem from condensation droplets), the total number of visible nodes and the proportion showing any retraction were assessed. Retraction was detected visually by sweeping across the time indicated when viewing the image stack on image j. The graph shows the scatter of the raw data: in some cases, data points lay on top of one another and, where this has happened, the red numbers in the graph indicate the number of points present. For times with more than one data point and with variation between them, the mean and standard deviation at that time are shown in the vertical text above the graph.

**Fig. 5:**
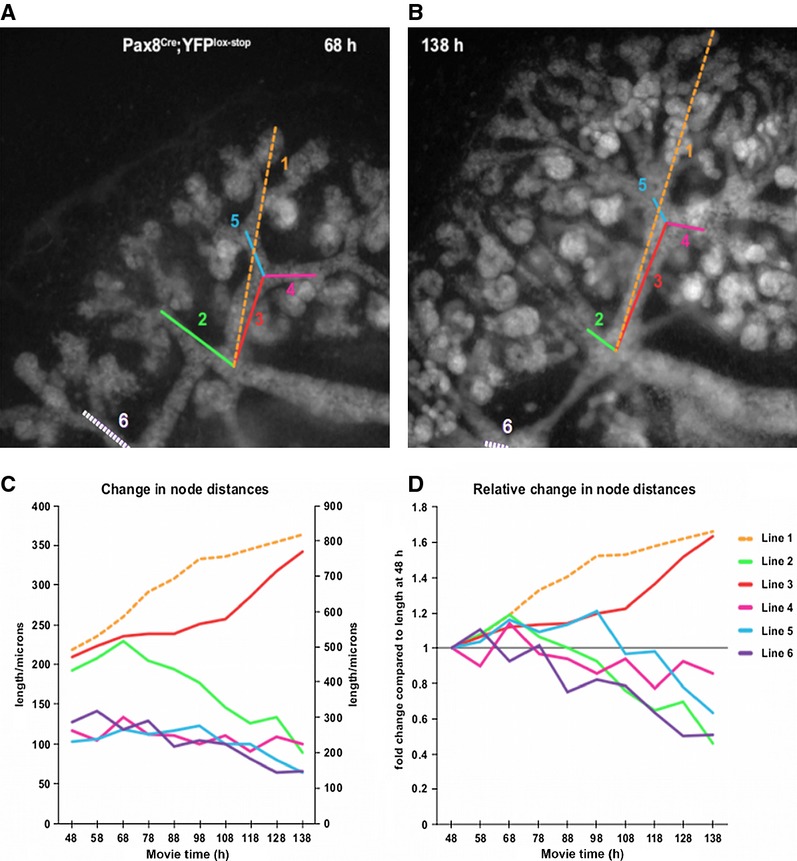
Node retraction in conventional (Saxen) culture. Panel (A) shows a portion of an E11.5 kidney that has been growing for 68 h in culture. The kidney is from a Pax8Cre;YFP^lox-stop^ mouse, in which both the developing collecting duct system and nephrons fluoresce. Panel (B) shows the same kidney 70 h later. Line 1 (dotted orange) indicates the distance from a particular branch node to the same tip in both images, and is used as a measure of radial tree growth. Lines 2–6 indicate the internodal lengths of four branches. Four of these, 2, 4, 5 and 6, show a net shortening by node retraction, whereas 3 shows a net elongation. The graphs (C,D) show the changing lengths of these same lines, identified by the same numbers and colours (except line 6, which is shown in purple in the graphs), in (C) absolute terms, and in (D) relative terms compared to their lengths in (A). In (C), the tree growth line, line 1, is plotted to the right Y-axis, while all internode lines are plotted to the left one. Scale: the frames are 385 μm across.

For time-lapse imaging for Fig. 5 and Supporting Information Movie S5, *Pax8*^+/Cre^ (Pax8^tm1(cre)Mbu^) mice (Bouchard et al. 2004) were crossed with *Rosa26*^eYFP/eYFP^ [Gt(ROSA)26^Sortm1(EYFP)Cos^] animals (Srinivas et al. 2001) to make *Pax8*^*Cre*^*GFP;YFP*^*lox-stop*^ animals. Three kidneys were isolated from *Pax8*^*Cre*^*GFP;YFP*^*lox-stop*^ embryos, placed on transwell filters (Millipore) and transferred to be imaged using an inverted Nikon TiE microscope, in humidified 5% CO_2_/air at 37 °C. Bright-field and YFP channels were captured every 20 min using a 4× objective. To reduce autofluorescence in the time-lapse experiments, the kidneys were cultured in phenol red-free media, supplemented with 10% fetal calf serum (FCS) and 1% penicillin/streptomycin. We have previously published an analysis of nephron formation using these *Pax8*^*Cre*^*GFP;YFP*^*lox-stop*^ kidneys (Lindström et al. 2013). Figure 4 was produced from analysis of four further wild-type kidneys imaged in bright field and three *TCF/Lef:H2B-EGFP* [Tg(TCF/Lef1-HIST1H2BB/EGFP)61Hadj] (Ferrer-Vaquer et al. 2010) × CD1 kidneys, the ureteric buds of which are marked with EGFP, were imaged using epifluorescence.

### Staining of newborn kidneys

Kidneys were isolated from newborn wild-type mice in cold PBS, then fixed for 1 h in 4% paraformaldehyde (PFA) at 4 °C. Kidneys were blotted dry, embedded in 15% gelatin (300 bloom) in cryomoulds, and allowed to set at 4 °C for 1 h. The gelatin blocks were removed from the moulds and fixed in 4% PFA overnight (18 h) at 4 °C. Following fixation, the blocks were washed three times in PBS for 5 min each, and then sectioned on a vibratome at 100 μm thickness. The sections were subjected to whole-mount *in situ* hybridization by a method described before (Yu et al. 2012) using a probe to Anapc11 (MGI Accession ID: MGI:3507268). Probe signal was detected with BM Purple (Roche).

### Analysis of movie images

For quantitative measurements, movie frames were printed out A4-sized, and length measurements were made on the printout using a ruler (this process was found to be more reproducible than using mouse and image j, probably because a clear ruler obscures details in the image much less than a mouse pointer). Internode lengths and pelvis-periphery lengths along the line of the branches were recorded (see Fig. 4). Each graph (Figs 1 and 5C,D) was made using measurement data from one single time-lapse movie, Fig. 1C being from a Sebinger culture and Fig. 5C and D from a Saxen culture.

### Computer modelling

Modelling was done using a simple algorithm to move tips and branch nodes on a 2-dimensional plane. The approach was very similar to that of Mandelbrot (1982) except that our model also included node retraction as a user-specified option. The model kept track of individual nodes and tips, including the lineage information about which node/tip was the daughter of which older node and the x,y position of each node on a 2-dimensional plane. Branches were depicted on images as straight lines connecting mother and daughter nodes/tips: the thickness of a branch line increased with the number of daughters it had (i.e. with age). Branches grew at a constant rate-per-unit-length. Retraction also occurred at a constant rate-per-unit-length but it was not applied to terminal branches. Every so often (at user-specified time intervals), tips bifurcated to produce two new daughters, the old tip becoming their mother node. The program began with an unbranched bud. Branches diverged at 93°, a value established from our own measurements of real cultured kidneys (Lindström N.O., Chang C.-H., Todd Valerius M., Hohenstein P., Davies J.A., unpublished data), which are very close to those reported by Watanabe & Costantini, 2004. Branching and growth stopped after a user-specified length of time. Retraction (if any) could continue for a user-specified period after this until the end of the run. The full source code, richly commented and with instructions for its use, is attached as Supporting Information Code S1.

### Ethics statement for use of experimental animals

Animal breeding was approved by the Edinburgh University Animal Welfare and Ethical Review Body. Transgenic animals were bred and kept at designated facilities at the University of Edinburgh and work was performed according to the regulations specified by the Home Office (UK) under the Project Licence 60/3788 to P.H.

## Results

### Kidney rudiments in low-volume culture show centripetal retraction of branch nodes

We have previously reported that E11.5 mouse kidney rudiments will, when cultured using the Sebinger culture system (Sebinger et al. 2010), develop an organotypic anatomy with distinct cortical and medullary zones (Sebinger et al. 2010) and loops of Henle (Chang and Davies, 2013). Nephron progenitors (cap mesenchyme, renal vesicles, comma- and S-shaped bodies) are restricted to the cortex, and almost all parts of more mature nephrons remain there: only the loops of Henle descend into the medulla. This means that, whereas the cortex is a crowded area as it is *in vivo*, the medulla is relatively empty and the medullary collecting duct system is particularly easy to observe.

When examining time-lapse images of kidneys that had been developing in this culture system for more than a week, we noticed a behaviour of maturing collecting ducts that seems not to have been described before: some of the the nodes (branching points) of the duct tree moved, over time, centripetally. This behaviour – ‘node retraction’ – can be seen by comparison of the movie frames in Fig. 1A, 9.4 days into culture, and Fig. 1B, 1.2 days later. Some nodes showed no movement during the period of the movie. At least nine nodes, in the half of the kidney captured in Fig. 1, did show movement: Fig. 1A′,B′ highlight these moving nodes. As can be seen clearly in Fig. 1B′, the retraction of the nodes was not merely a retraction relative to the overall size of the kidney (e.g. a ‘failure to keep up’). Rather, it was an absolute movement in space, so that the branch leading to a node became shorter. Nodes did not all retract at the same time and the rate, both absolute and relative to original internode length, varied between nodes at one time, and for the same node over time (Fig. 1C).

The retraction of a node took place without significant circumferential movement of the ends of its daughter branches. This had the effect of making the angle between these daughter branches more acute. This effect can be seen in the high-magnification view in Fig. 2. The node marked ‘i’ in the skeletonized tracing initially had daughter branches that diverged at about 75°: 1.2 days later, after node retraction, the basal parts of these branches ran almost parallel, and even when the branches finally diverged, they did so at a much more acute angle.

While some nodes were retracting, the more basal branches towards which the nodes were heading often became visibly broader: this can be seen in Fig. 3A,B, in which diameter lines that span the branches precisely in Fig. 3A are superimposed on the later frame B, and can be seen to be too short to span the now wider tubes. The time-lapse images from which these frames were taken can be viewed as Supporting Information Movies S0–S2.

### Node retraction is also seen in conventional (Saxen) organ culture systems

Kidney rudiments are traditionally grown on polycarbonate filters on the surface of large volumes (≥ 1 mL) of medium, supported either by a Trowell screen or a well insert. To determine whether node retraction is also a feature of this culture system, ‘Saxen culture’, and therefore not an artefact of the Sebinger culture system, we looked for evidence for node retraction in time-lapse studies of seven separate Saxen cultures, all set up from E12.5 to E12.75 embryos. There was no evidence of retraction in the first 2 days of culture. The first, weak examples appeared at 2.5 days of culture, with around 1/5 of visible nodes retracting by 4.5 days of culture (Fig. 4). Longer-term culture did not show the very clear zoning into cortex and medulla seen in Sebinger cultures. Nevertheless, node retraction was shown as clearly as in Sebinger cultures. Figure 5A,B shows high-magnification views of the edge of a growing kidney of a *Pax8*^*Cre*^*GFP;YFP*^*lox-stop*^ mouse. The kidney was still growing in overall diameter at this stage, but at least four nodes in the images, at the ends of the branches labelled ‘2’, ‘4’, ‘5’ and ‘6’, were retracting. At the same time, one branch (‘3’) was still growing. The nodes moved centripetally in both absolute and relative terms (Fig. 5C). Figure 5C,D, which represents measurements of these branches in the same kidney, illustrates this phenomenon. This suggests that retraction is something that occurs on a node-by-node basis and not in response to an organ-wide event. Retraction could be seen even in young nodes near the outside of the kidney as well as in older ones near the middle: an example of an outer node retracting is shown in the top right, ringed, area of the file ‘Movie S5 Annotated’.

### Node retraction can, in principle, turn a fractal tree into a more organotypic one

**Fig. 6:**
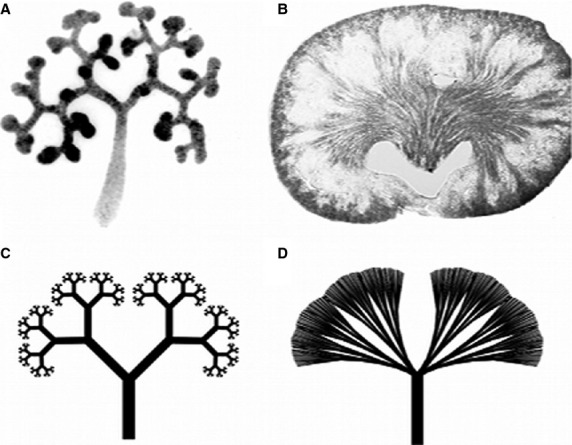
Branching models without node retraction generate trees similar to the ureteric buds of young kidney rudiments, whereas the same models with node retraction added generate trees more similar to those of mature collecting ducts. (A) Ureteric bud tree of an E11.5 kidney cultured for 72 h and stained with anti-calbindin-D28k to show the ureteric bud. (B) The collecting ducts in a portion of a newborn mouse kidney stained for *Anapc11* mRNA which, though not exlclusive to collecting ducts, shows the medullary ducts very clearly. Images (C) and (D) show the result of a simple computer model of branching, run either without any node retraction (C), which produces a spread tree, like (A), or with node retraction applying to all nodes except the central one (the ‘pelvis’) and the terminal tips. Node retraction produces long, radial medullary ducts reminiscent of those in (B): the model does not include pelvic enlargement (the mechanism for which is not yet understood) so, in the model, branches eventually converge on a normal-sized trunk, not a wide pelvis. The numbers of tips are the same in (C) and (D).

Mathematical treatments of branching morphogenesis typically assume a process of repeated branching, with all existing branches elongating steadily. This approach, which can be traced back at least to Leornardo da Vinci (Long, 2004), famously appears in Benoit Mandelbrot's *Fractal Geometry of Nature* (Mandelbrot, 1982), in which it is used to generate an idealized lung. We have implemented a model using this principle, but have altered it to use a 90° divergence angle rather than an 180 ° one, for the sake of making it more realistic for kidney (a brief explanation of the working of the model appears in Materials and Methods and full source code is available as Supporting Information Code S1). Run with regular bifurcation and constant growth per unit length, as would emerge from uniform rates of cell division, with some width expansion of tubes as well, the model generates a self-similar (fractal) tree (Fig. 6C) that is reminiscent of the ureteric bud/collecting duct anatomy of a living young kidney rudiment (Fig. 6A). This fractal-like anatomy, while realistic for immature kidneys, is very different from the anatomy of the mature collecting duct tree, in which branches diverge in the deep medulla and run as almost parallel radial spokes up towards the cortex (Fig. 6B).

It is possible to run the computer model that generated Fig. 6C, so that nodes are subject to retraction as well as growth (in the model, branch nodes are subject to retraction but the tips of the tree are not). When this alteration is made, the tree generated is of a different shape. In particular, if retraction is allowed to continue for some time after new growth and branching has ceased, the algorithm generates trees that feature widened deep branches and long, thin radial branches that run almost parallel to one another (Fig. 6D). It also organizes the branches into distinct groups, suggestive of the (more numerous) ‘pyramids’ of a human kidney.

Simple mathematical models such as this one are simply exploratory tools and are not intended to be faithful representations of the working of an actual organ. Nevertheless, the model does suggest a plausible function for the node retraction we have observed in movies of real cultured kidneys (see Discussion below).

## Discussion

The branching pattern of the urinary collecting duct system changes during renal development. It begins as a simple fractal-like tree in which branches spread out and branch nodes are present throughout the structure, being particularly common towards the outside of the organ (simply because the tree extends outwards and the number of nodes doubles with each generation of branching). By the time the kidney has matured, the tree has a very different shape. Its deepest parts form a very enlarged renal pelvis. In the medulla, collecting duct branches run radially, almost parallel to one another, and do not connect via nodes scattered richly in the outer kidney but rather converge in the papilla regions immediately outside the pelvis. In this report, we have provided evidence from movies of cultured mouse kidney rudiments that nodes of the collecting duct tree can retract towards what would be the developing renal pelvis, and suggest that this mechanism may account, at least in part, for the change in pattern of the tree.

The transition from the early pattern to the mature one has so far attracted little research attention, and much of what has been written was written long ago, based on interpretation of static images. Edith Potter provided the best classical descriptions of renal morphogenesis, based on careful micro-dissection. In her book (Potter, 1972) she did not describe node retraction as such, but she did note that, where a collecting duct branch in a maturing kidney is unusually short, a ‘compensatory change in length’ occurs in its daughter branches. In our time-lapse studies, retraction of a mother node leaves the daughter branches longer than they were, so that the same net distance is spanned (see, for example, the branch between nodes i and ii in Fig. 2), matching Potter's description. Potter also noted the expansion in girth of early generation tubules to make the renal pelvis and calyces, but considered outward expansion of the dilated area of calyces to be the explanation for many collecting ducts feeding into the one calyx rather than still connecting sequentially in a tree-like way. Also working from fixed samples of different ages, Cebrián et al. (2004) have provided a detailed morphometric study of mouse kidney development. They draw attention to two phases of ureteric bud/collecting duct morphogenesis, one being early-type branching and the other, after E15.5, involving a marked increase of the distance between cortical branches and the deep medulla/pelvis. In their discussion, they propose that internodal growth of specific branches may account for this, and point out that the elongation coincides with the differentiation of renal stroma. Recent reviews of collecting duct morphogenesis (Costantini & Kopan, 2010; Costantini, 2012) have assumed that the transition from the immature shape to the long, radial medullary tube form relies on orientated cell division and convergent extension.

That node retraction has not been described from static images is not surprising, as it can only be seen properly by examination of the same nodes at different times. Watanabe and Costantini's pioneering time-lapse study of GFP-reporter collecting duct development in culture (Watanabe & Costantini, 2004) provided much valuable information about the dynamics of branching, but did not report node retraction, probably because it did not run long enough. The culture period, starting with E11.5 kidneys, ended at 70 h, by which time the elongation of older branches had ended but actual retraction had not begun. The group's subsequent original publications (Chi et al. 2009; Willecke et al. 2011) have also concentrated on the first 72 h of development from E11.5, which is an ideal time for studying mechanisms of branching but is too early for retraction.

What might control node retraction? The fact that not all nodes retract simultaneously suggests that the phenomenon cannot be triggered solely by a global, organ-wide signal. Cebrián et al. (2004) noted that the transition from early-pattern branching to having long medullary ducts coincided with differentiation of the stroma, and pointed out that mutants that inhibit stromal development have an abnormally shallow medulla. It has been reported that the secreted BMP inhibitor *Cer1* increases the total size of a collecting duct tree but, paradoxically, reduces the distance between the renal pelvis and the first distinct collecting duct branching point (Chi et al. 2011: see their Fig. S5 for the pelvis-to-branch data). The peculiar phenotype could be explained if stroma-derived BMPs play a role in regulating node retraction (positively or negatively: the abnormally close nodes in Chi et al. could be ones that were meant to retract all the way to the pelvis and failed to do so, or younger ones that have retracted too far from the upper medulla). It is possible, even in normal development, that the choice between a node reaching and being absorbed into the pelvis or remaining intact in the cortex or medulla simply reflects whether the node has time to retract all the way, before the era of retraction ends.

Although it has not been described before in epithelia, a process that is at least superficially analogous has been described in vascular remodelling on the chicken chorioallantoic membrane. Here, node retraction occurs by intussusceptive branch remodelling, the details of which have been deduced by scanning electron microscopy of vascular casts (Djonov et al. 2002). A short distance down the stem of the ‘Y’ of a branch point, invaginations from the top and bottom surfaces of the vessel grow inwards and meet to make a pillar. The pillar then expands towards the open end of the ‘Y’, eventually reaching the surrounding mesenchyme so that the branch point has moved to the original position of the pillar. Repeated iterations of this mechanism can cause the branch node to travel a long way, and the angle of divergence to narrow considerably (thus reducing blood turbulence). Time-lapse movies of this vessel remodelling (Djonov et al. 2002) look, in terms of tree anatomy, very similar to the node retraction we have discovered in the collecting ducts. Our movies do not, however, show any evidence of intussusceptive invagination of pillars.

Our observations on living kidney rudiments suggest that node retraction is one of the morphogenetic processes involved in maturation of the collecting duct system. Our simple computer models have also suggested that this mechanism might be helpful in converting the early tree to its mature form. Node retraction may link to the orientated cell division that has been assumed to drive branch elongation (Costantini & Kopan, 2010; Costantini, 2012). If elongation were driven by cell division within a tube, though, the longitudinal forces would be compressive, tending to bend or buckle the tube. It is noticeable that medullary collecting ducts run very straight as they elongate distal to a retracting node, and straightness suggests tension. Over a century ago, Oscar Hertwig (1893) noted that tissues subject to tension tended to orientate their mitoses in a direction that would reduce that tension (see Davies, 2013 for a review). It is therefore possible that tension developed by node retraction helps to orientate the cell division of overlying branches, and that orientated cell division is therefore an effect, rather than a cause, of tree remodelling.

Whatever its mechanism, we suggest that node retraction might be an important morphogenetic tool used in the development of the kidney. There remains, however, the caveat that our observations were made only in culture (making time-lapse studies of kidneys growing *in vivo* deep inside a mother mouse, with the organs having to remain completely still, over several days, is simply not feasible). It is possible that the mechanical influence of the substrate interacts with normal biophysical features of renal development to create node retraction artefactually. We view this as unlikely, given how well node retraction can explain the transformation shape of the collecting duct tree, but we acknowledge that there is room for debate. We therefore write this report in the spirit of an intriguing observation that might stimulate future research, rather than a definitive proof.

## Supporting Information

**Code S1.** Code for the computer simulation.

**Movie S0.** Wide-field view of the Sebinger-culture movie analysed in closer detail in Movies S1–S2 and Figs 1–2 in the main text.

**Movie S0 Annotated.** This is identical to Movie S0, with the addition of red rings to highlight some nodes showing retraction, and blue rings to highlight some collecting ducts downstream of retracting nodes that show expansion in girth.

**Movie S1.** Movie that is the source of the still images shown in Fig. 1.

**Movie S2.** Movie that is the source of the still images shown in Fig. 2. (There is no movie S3 or S4, because the movies have been numbered to match the main-text figures created from them).

**Movie S5.** Movie analysed in Fig. 4 of the main text: this culture is from Saxen culture, not Sebinger culture.

**Movie S5 Annotated.** Movie S5 (inverted to be black-on-white for clarity), with two areas of interest circled.

**Movie S5 Examples.** Start and end frames of Movie S4 Annotated, with specific nodes marked with arrows on both frames, to illustrate movement.
