# Supplementary material for: Node retraction during patterning of the urinary collecting duct system
Source: J Anat. 2014 Oct 7;226(1):13–21. doi: 10.1111/joa.12239 (PMC4299504; doi:10.1111/joa.12239)

MovieS4: examples of retracting node.

This shows the start and end frames of MovieS4Annotated.avi, with two examples of retracting nodes indicated in their start and end positions, by arrows. The image is inverted (black-on-white) because it is easier to see when printed.

The faint red rings are in the same place

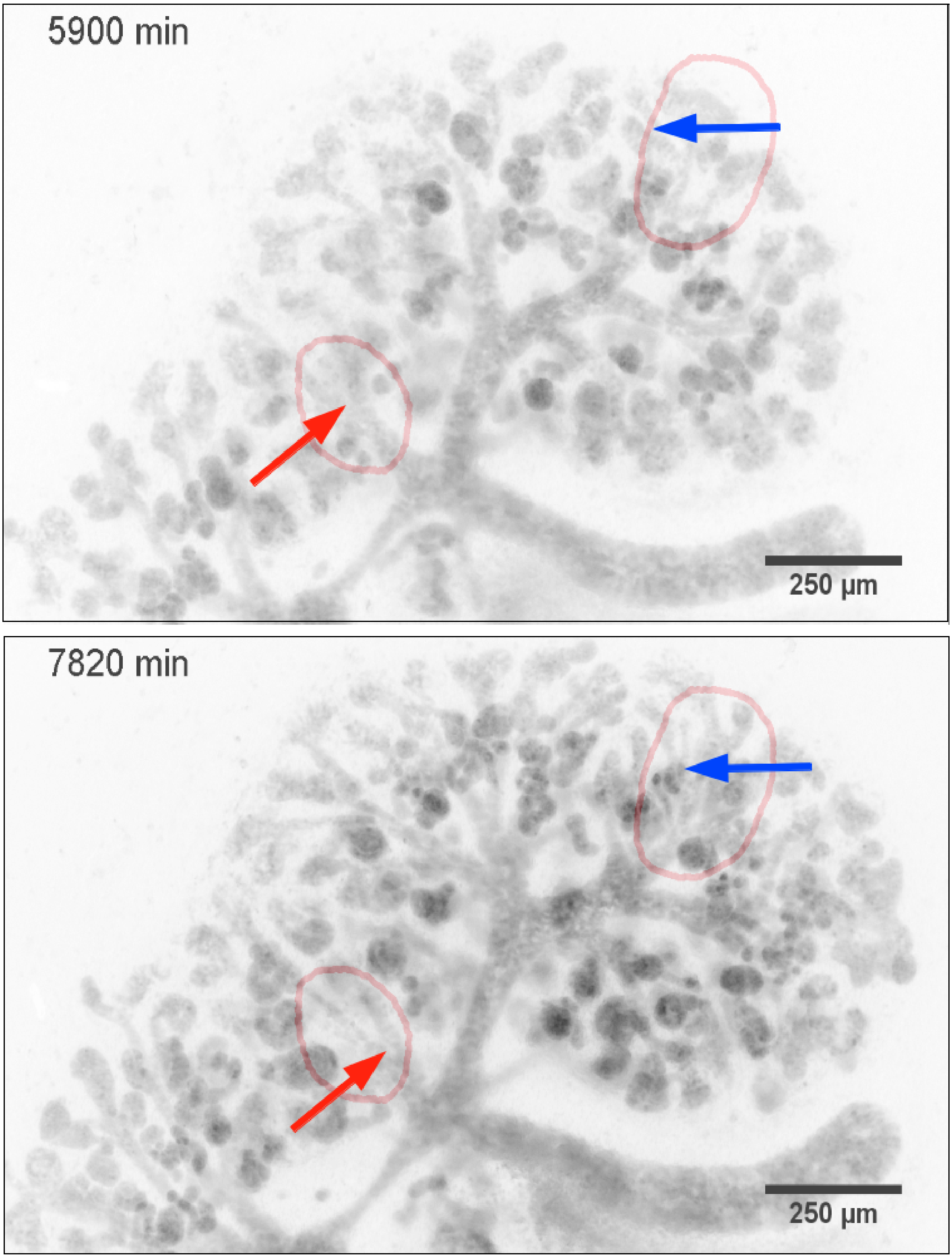

Supplement: Supplementary file 9 — Movie S5 Examples. Start and end frames of Movie S4 Annotated, with specific nodes marked with arrows on both frames, to illustrate movement. [file joa0226-0013-sd9.pdf]
