# Supplementary figures and images for: Node retraction during patterning of the urinary collecting duct system
Source: J Anat. 2014 Oct 7;226(1):13–21. doi: 10.1111/joa.12239 (PMC4299504; doi:10.1111/joa.12239)

Key to the lengths measured in Fig 1c:

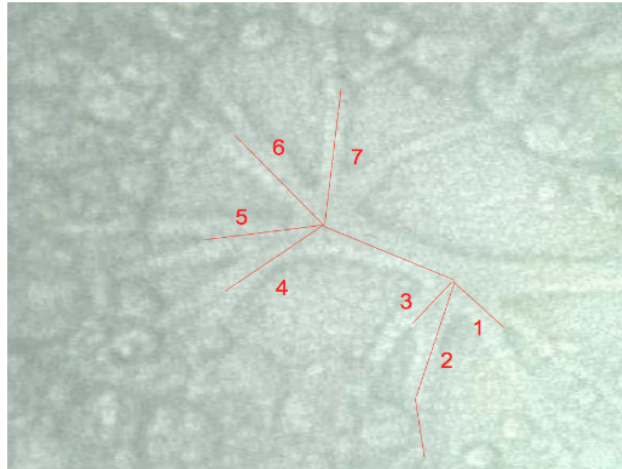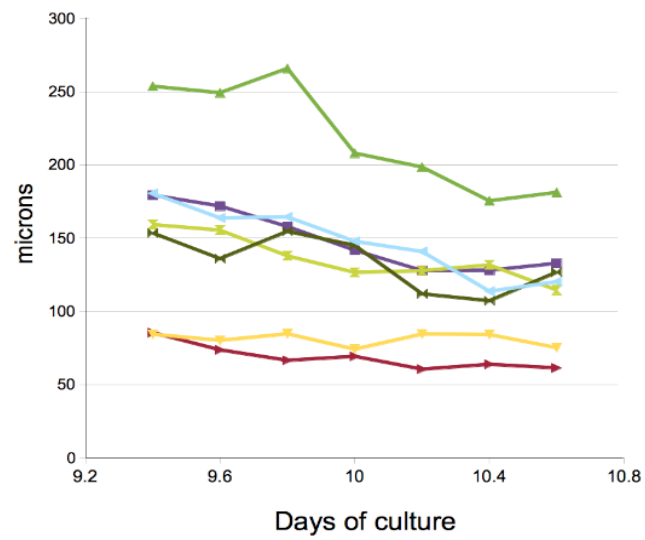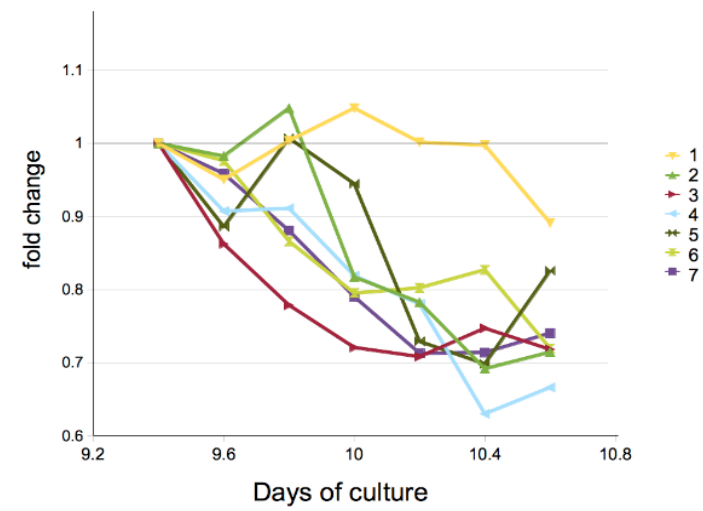

Supplement: Supplementary file 10 — Movie S5 Examples. Start and end frames of Movie S4 Annotated, with specific nodes marked with arrows on both frames, to illustrate movement. [file joa0226-0013-sd10.pdf]
