# Supplementary material for: Node retraction during patterning of the urinary collecting duct system
Source: J Anat. 2014 Oct 7;226(1):13–21. doi: 10.1111/joa.12239 (PMC4299504; doi:10.1111/joa.12239)
Supplement: Supplementary file 11 — Movie S5 Examples. Start and end frames of Movie S4 Annotated, with specific nodes marked with arrows on both frames, to illustrate movement. [file joa0226-0013-sd11.pdf]

Guide to Supplementary Information

Code S1: this is the code for the computer simulation. It is written in the language Processing. The easiest way to run it is to launch Processing, open a new window, and paste the text of CodeS1 in (this way, Processing will automatically put it in an appropriate directory: the file can be run directly from the .pde file, but some implementations of Processing will object if this file has not been put not in the correct Processing subdirectory before being opened).

Movie S0: This is the wide-field view of the Sebinger-culture movie analysed in closer detail in Movies S1-S2 and Figures 1-2 in the main manuscript. No direct measurement were made from this movie – it is provided simply in the spirit of absolute transparency, to show raw, uncropped data both in the original colour form and the monochrome that used to generate the detailed movies S1-S2.

MovieS0Annotated: This is identical to Movie S0, with the addition of red rings to highlight some nodes showing retraction, and blue rings to highlight some collecting ducts downstream of retracting nodes, that show expansion in girth.

Movie S1: this is the movie that is the source of the still images shown in Fig 1.

Movie S2: this is the movie that is the source of the still images shown in Fig 2.

(There is no movie S3 of S4, because the movies have been numbered to match the main-text figures created from them).

Movie S5: this is the movie analysed in Fig 4 of the main text: this culture is from Saxen culture, not Sebinger culture.

Movie S5 Annotated: this is movie S5 (inverted to be black-on-white for clarity), with two areas of interest circled. **Movie S5 Examples** shows the start and end frames of Movie S4 Annotated, with specific nodes marked with arrows on both frames, to illustrate movement.
